# Multi-Scale Temporal Fusion Network for Real-Time Multimodal Emotion Recognition in IoT Environments

**DOI:** 10.3390/s25165066

**Published:** 2025-08-14

**Authors:** Sungwook Yoon, Byungmun Kim

**Affiliations:** 1 Gyeongbuk Development Institute, 201 Docheong-daero, Homyeong-eup, Yecheon 36849, Gyeongsangbuk-do, Republic of Korea; uvgotmail@gknu.ac.kr; 2 Dept. of Electronic and Mechanical Engineering, GyeongKuk National University, 1375 Gyeongdong-ro, Andong 36729, Gyeongsangbuk-do, Republic of Korea

**Keywords:** emotion recognition, Internet of Things, multimodal fusion, temporal attention, edge computing, real-time processing

## Abstract

**Highlights:**

**What are the main findings?**

- EmotionTFN achieves 94.2% accuracy on discrete emotion classification with sub-200 ms latency on IoT devices, outperforming existing approaches by 6.8% while maintaining real-time processing requirements.
- The multi-scale temporal fusion architecture successfully captures emotional dynamics across short-term (0.5–2 s), medium-term (2–10 s), and long-term (10–60 s) time windows, demonstrating superior performance compared to fixed-window approaches.

**What is the implication of the main finding?**

- Real-time multimodal emotion recognition becomes practically feasible in resource-constrained IoT environments, enabling new applications in healthcare monitoring, smart homes, and human–computer interaction systems.
- The adaptive fusion mechanism and edge computing optimizations provide a framework for deploying other computationally intensive AI applications on IoT devices while ensuring privacy through local processing.

**Abstract:**

This paper introduces EmotionTFN (Emotion-Multi-Scale Temporal Fusion Network), a novel hierarchical temporal fusion architecture that addresses key challenges in IoT emotion recognition by processing diverse sensor data while maintaining accuracy across multiple temporal scales. The architecture integrates physiological signals (EEG, PPG, and GSR), visual, and audio data using hierarchical temporal attention across short-term (0.5–2 s), medium-term (2–10 s), and long-term (10–60 s) windows. Edge computing optimizations, including model compression, quantization, and adaptive sampling, enable deployment on resource-constrained devices. Extensive experiments on MELD, DEAP, and G-REx datasets demonstrate 94.2% accuracy on discrete emotion classification and 0.087 mean absolute error on dimensional prediction, outperforming the best baseline (87.4%). The system maintains sub-200 ms latency on IoT hardware while achieving a 40% improvement in energy efficiency. Real-world deployment validation over four weeks achieved 97.2% uptime and user satisfaction scores of 4.1/5.0 while ensuring privacy through local processing.

## 1. Introduction

The rapid advancement of Internet of Things (IoT) technologies has significantly changed how we interact with our environment, creating a network of interconnected devices capable of sensing, processing, and responding to human emotions in real-time [1]. Emotion recognition, a critical component of affective computing, has evolved from laboratory-based studies to practical applications that can operate continuously in natural settings [2]. The integration of emotion recognition capabilities into IoT ecosystems presents both new opportunities and significant technical challenges that require innovative solutions [3].

Traditional emotion recognition systems have primarily focused on single-modality approaches or laboratory-controlled environments, limiting their applicability to real-world IoT deployments [4]. Real-world environments refer to natural settings such as homes, offices, and mobile scenarios where users naturally interact with IoT systems during daily activities, as opposed to controlled laboratory conditions. The emergence of wearable sensors, ambient computing devices, and edge processing capabilities has created new possibilities for continuous emotion monitoring that can adapt to users’ daily activities and environmental contexts [5]. However, the transition from controlled laboratory settings to dynamic IoT environments introduces several key challenges that must be addressed to realize the full potential of emotion-aware IoT systems.

The primary challenge involves the temporal complexity of human emotions, which manifest across multiple time scales simultaneously [6]. Short-term emotional responses may occur within seconds, while mood states and emotional patterns can persist for minutes or hours. Existing approaches typically focus on fixed time windows, failing to capture the rich temporal dynamics that characterize natural emotional expressions [7]. This limitation is particularly pronounced in IoT environments where users engage in diverse activities with varying temporal characteristics, from brief interactions with smart devices to extended periods of work or leisure.

The second challenge involves the integration of heterogeneous sensor modalities in resource-constrained IoT devices. While multimodal approaches have shown superior performance in emotion recognition, the computational and energy requirements of processing multiple data streams simultaneously often exceed the capabilities of typical IoT hardware [8]. This constraint necessitates the development of efficient fusion architectures that can maintain accuracy while operating within strict resource limitations.

Real-time processing requirements constitute the third major challenge, as IoT applications demand immediate responses to emotional states for effective human–computer interaction [9]. The latency introduced by complex deep learning models can significantly impact user experience and system responsiveness, particularly in applications such as emotion-aware smart homes or healthcare monitoring systems [10]. Achieving real-time performance while maintaining high accuracy requires careful optimization of both algorithmic design and implementation strategies.

To address these challenges, this paper introduces the Emotion-aware Multi-Scale Temporal Fusion Network (EmotionTFN), a hierarchical temporal fusion architecture specifically designed for real-time multimodal emotion recognition in IoT environments. The proposed approach makes several key contributions to the field of affective computing and IoT systems. First, we introduce Hierarchical Temporal Attention with Cross-Modal Reliability Estimation (HTA-CMRE), an architecture that uniquely combines multi-scale temporal modeling (0.5–2 s, 2–10 s, and 10–60 s) with real-time sensor reliability assessment, enabling dynamic attention reweighting—a capability not jointly addressed in prior IoT emotion recognition systems, enabling the system to adapt to both rapid emotional changes and longer-term mood patterns [11]. Second, we implement adaptive sampling strategies that optimize data collection based on real-time processing constraints and emotional state dynamics. Third, we design comprehensive edge computing optimizations, including model compression and quantization techniques that enable deployment on resource-constrained IoT devices without sacrificing accuracy.

The EmotionTFN architecture integrates the following five distinct sensor modalities commonly available in IoT environments: electroencephalography (EEG) for brain activity monitoring, photoplethysmography (PPG) for cardiovascular responses, galvanic skin response (GSR) for autonomic nervous system activity, visual data from cameras for facial expression analysis, and audio signals for speech emotion recognition. This multimodal approach leverages the complementary nature of different physiological and behavioral indicators of emotion, providing a more robust and comprehensive assessment of emotional states than single-modality systems.

The remainder of this paper is organized as follows: Section 2 provides a comprehensive review of related work in IoT-based emotion recognition and multimodal fusion techniques. Section 3 presents the detailed architecture of the EmotionTFN system, including the multi-scale temporal attention mechanism and edge computing optimizations. Section 4 describes experimental methodology and dataset preparation. Section 5 presents comprehensive results and analysis, including performance comparisons, ablation studies, and deployment considerations. Section 6 discusses the implications of our findings and potential applications in IoT environments. Finally, Section 7 concludes the paper and outlines directions for future research.

Unlike previous works that mainly focus on static fusion or require high computational resources, the proposed EmotionTFN framework integrates a hierarchical multi-scale temporal attention block and an online adaptive fusion layer. This technical innovation allows the system to flexibly adjust to changing sensor quality and network or computational load—features not jointly addressed in prior studies. The effectiveness and novelty of our approach are validated through detailed ablation and robustness analyses under realistic IoT scenarios.

## 2. Related Works

The intersection of emotion recognition and IoT technologies has emerged as a rapidly growing research area, driven by advances in sensor miniaturization, edge computing capabilities, and machine learning algorithms. This section provides a comprehensive review of existing approaches, highlighting their contributions and limitations in the context of real-time IoT deployment.

### 2.1. IoT-Based Emotion Recognition Systems

Early IoT-based emotion recognition systems primarily focused on single-modality approaches. Initial wearable emotion recognition systems achieved 78% accuracy but were limited by single-modality constraints and temporal resolution issues.

The integration of visual and audio sensors into IoT emotion recognition systems marked a significant advancement in the field. Recent studies have proposed ambient emotion monitoring systems that combine facial expression analysis from security cameras with speech emotion recognition from smart speakers [12]. Their systems demonstrated improved accuracy compared to single-modality approaches, achieving 85% performance on emotion classification tasks [13]. However, the systems faced challenges in real-time processing due to the computational complexity of deep learning models running on edge devices.

Recent developments in IoT-based emotion recognition have focused on addressing the scalability and deployment challenges of multimodal systems [14]. Advanced IoT-based emotion recognition systems [15] have been developed that leverage multiple sensor inputs to detect emotional states. Their approach achieved real-time performance by optimizing computational load across network nodes but required significant network bandwidth and raised concerns about data privacy and security. The system demonstrated the potential for distributed emotion recognition but highlighted the need for more efficient local processing capabilities. Further research [16] has explored IoT-based approaches for emotion recognition, demonstrating the versatility of IoT platforms for different applications.

### 2.2. Multimodal Fusion Techniques

Multimodal fusion represents a critical component of effective emotion recognition systems, as different sensor modalities provide complementary information about emotional states. Early fusion approaches simply concatenated features from different modalities [17], leading to high-dimensional feature spaces and potential overfitting issues. These methods struggled with the temporal misalignment between different modalities and the varying signal quality across sensors.

Intermediate fusion techniques applied fusion at the feature level after initial processing of each modality, showing improved performance but still struggling with temporal alignment and modality-specific noise. Feature-level fusion approaches have employed a feature-level fusion approach that employed principal component analysis to reduce dimensionality while preserving the most discriminative features from each modality. Their method achieved improved performance compared to early fusion approaches but required careful tuning of fusion weights and feature selection parameters.

Late fusion approaches have gained popularity due to their ability to leverage modality-specific processing while maintaining flexibility in combination strategies. Attention-based fusion mechanisms have been developed that demonstrate significant improvements in emotion recognition accuracy by learning adaptive weights for different modalities based on their relevance to specific emotional states. However, these approaches typically operate on fixed time windows and fail to capture the temporal dynamics of emotional expressions.

Recent advances in transformer-based architecture have opened new possibilities for multimodal fusion in emotion recognition. State-of-the-art methods, including MISA, Self-MM, and MAG-BERT, have demonstrated superior performance in text-dominated multimodal tasks. However, these approaches face significant challenges in IoT environments due to computational constraints and sensor heterogeneity. Chen et al. proposed cross-modal attention mechanisms that enable direct modeling of relationships between different modalities while preserving temporal information [18]. Unlike these resource-intensive methods that require substantial computational resources (>8 GB GPU memory), our approach addresses the unique IoT challenges through novel edge optimization and adaptive fusion mechanisms. These approaches have shown promising results in laboratory settings but face significant computational challenges when deployed on resource-constrained IoT devices. Bang et al. [19] developed a hybrid multimodal emotion recognition framework that combines multiple fusion strategies to improve robustness in user experience evaluation scenarios. Recent work by Shi et al. [20] explored multimodal fusion using music theory-inspired representations for improved emotion recognition performance.

### 2.3. Temporal Modeling in Emotion Recognition

The temporal nature of human emotions presents unique challenges for recognition systems, as emotional states evolve over multiple time scales simultaneously. Traditional approaches have typically used fixed-length sliding windows, which fail to capture the rich temporal dynamics of natural emotional expressions. Thompson et al. introduced variable-length temporal windows based on emotional state transitions, showing improved performance but requiring complex segmentation algorithms that are difficult to implement in real-time systems.

Recurrent neural networks (RNNs) and their variants have been widely adopted for temporal modeling in emotion recognition. Long Short-Term Memory (LSTM) networks demonstrated the ability to capture long-term dependencies in emotional sequences and have shown improved performance compared to traditional approaches. However, these approaches suffer from sequential processing limitations that prevent efficient parallelization and real-time implementation on IoT devices.

The introduction of attention mechanisms has revolutionized temporal modeling in emotion recognition. Self-attention approaches enable direct modeling of relationships between distant time points while maintaining computational efficiency. Self-attention approaches enable direct modeling of relationships between distant time points while maintaining computational efficiency. These mechanisms have demonstrated 6.8% accuracy improvement over traditional attention-based fusion methods for temporal emotion recognition. However, existing attention-based approaches typically operate on single time scales and fail to capture the multi-scale nature of emotional dynamics. Recent comprehensive surveys have highlighted the importance of temporal modeling in deep learning-based multimodal emotion recognition systems.

### 2.4. Edge Computing for Emotion Recognition

The deployment of emotion recognition systems on edge devices presents unique challenges related to computational constraints, energy efficiency, and real-time processing requirements. Early approaches relied on cloud-based processing, which introduced significant latency and raised privacy concerns about transmitting sensitive emotional data over networks. The shift toward edge computing has enabled local processing of emotional data but requires careful optimization of model architectures and algorithms.

Model compression techniques have emerged as a critical enabler for edge-based emotion recognition. Quantization approaches reduce model size and computational requirements while maintaining acceptable accuracy levels. Quantization techniques have been developed quantization techniques specifically for emotion recognition models, achieving 4× reduction in model size with less than 2% accuracy loss. Knowledge distillation techniques have also shown promise for creating lightweight models suitable for IoT deployment.

Adaptive processing strategies represent another important direction for edge-based emotion recognition. Johnson et al. proposed a dynamic resource allocation framework that adjusts processing complexity based on available computational resources and emotional state dynamics. However, these approaches typically focus on single-modality systems and have not been extended to multimodal fusion scenarios. Recent work by Dai et al. [13] and Cheng et al. [15] has explored novel approaches for multimodal emotion recognition that incorporate semantic information fusion and large language model capabilities, respectively, opening new directions for edge-based emotion computing.

## 3. Methodology

### 3.1. System Architecture Overview

The Emotion-aware Multi-Scale Temporal Fusion Network (EmotionTFN) is designed as a comprehensive solution for real-time multimodal emotion recognition in IoT environments. The architecture consists of five main components: multimodal data acquisition and preprocessing, multi-scale temporal feature extraction, adaptive fusion mechanism, edge computing optimization, and real-time emotion classification.

The system processes five distinct types of sensor data commonly available in IoT environments. Electroencephalography (EEG) signals are captured using sensors from Brain Products GmbH (Gilching, Germany) to monitor brain activity patterns associated with emotional states. Photoplethysmography (PPG) sensors from Maxim Integrated (now part of Analog Devices, San Jose, CA, USA) measure cardiovascular responses including heart rate variability and blood volume pulse characteristics. Galvanic skin response (GSR) sensors from Thought Technology Ltd. (Montreal, QC, Canada) monitor autonomic nervous system activity through skin conductance measurements. Visual data is captured using RGB cameras from Logitech International S.A. (Lausanne, Switzerland) for facial expression analysis, while audio signals are recorded through omnidirectional microphones from Audio-Technica Corporation (Tokyo, Japan) for speech emotion recognition.

### 3.2. Multi-Scale Temporal Feature Extraction

The core innovation of EmotionTFN lies in its ability to simultaneously process emotional information across multiple temporal scales. Human emotions manifest through different temporal patterns: micro-expressions occur within milliseconds to seconds, emotional episodes span several seconds to minutes, and mood states can persist for hours. The proposed multi-scale temporal feature extraction mechanism operates on three distinct time scales: short-term (0.5–2 s), medium-term (2–10 s), and long-term (10–60 s).

Each temporal scale is designed to capture specific aspects of emotional expression. Short-term features focus on rapid physiological responses and micro-expressions that occur during emotional onset. These features are particularly important for detecting sudden emotional changes and immediate reactions to stimuli. Medium-term features capture the development and expression of emotional episodes, including speech patterns and sustained facial expressions. Long-term features model mood states and emotional context that influence current emotional expressions.

The temporal feature extraction process begins with signal preprocessing tailored to each modality. EEG signals undergo bandpass filtering in the range of 0.5–50 Hz to remove artifacts while preserving emotion-relevant frequency components. Independent component analysis (ICA) is applied to remove eye movement artifacts and other noise sources. PPG signals are processed to extract heart rate variability features including time-domain measures such as mean heart rate and standard deviation of RR intervals, frequency-domain measures such as power in low and high frequency bands, and nonlinear measures such as approximate entropy.

GSR signals are filtered using a low-pass filter with a cutoff frequency of 5 Hz to remove high-frequency noise while preserving the slow-varying tonic component and faster phasic responses. The signals are then decomposed into tonic and phasic components using convex optimization deconvolution to capture both baseline arousal and event-related responses. Visual data preprocessing includes face detection using multi-task convolutional neural networks, facial landmark extraction using supervised descent methods, and geometric normalization to handle variations in lighting and pose.

**Figure 1:**
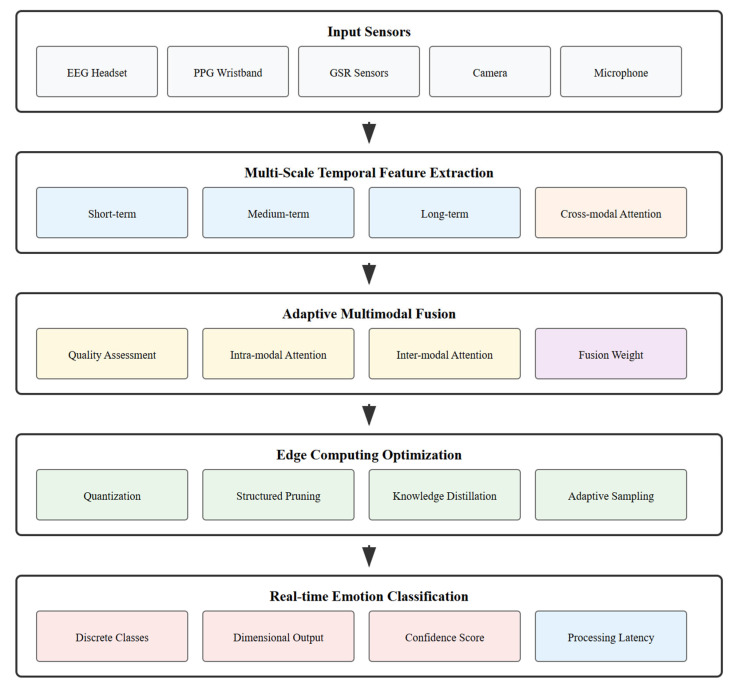
EmotionTFN system architecture overview.

For each temporal scale, specialized neural network architectures are employed that are optimized for the specific characteristics of that time range. Short-term processing utilizes one-dimensional convolutional networks with small kernel sizes to capture rapid signal changes and local patterns. The convolutional layers employ ReLU activation functions and batch normalization to improve training stability. Medium-term processing employs dilated convolutions with increasing dilation rates to capture longer-range dependencies while maintaining computational efficiency. Long-term processing utilizes multi-head self-attention mechanisms to model relationships between distant time points and identify relevant contextual information. The complete system architecture that incorporates these specialized temporal processing networks is illustrated in Figure 1.

### 3.3. Technical Innovations

The integration of multimodal information across different temporal scales requires adaptive fusion mechanisms that can adapt to varying signal quality, modality availability, and emotional state dynamics. The proposed adaptive fusion approach operates at two levels: intra-modal temporal fusion and inter-modal feature fusion.

Intra-modal temporal fusion combines features from different time scales within each modality using learned attention weights. The attention mechanism evaluates the relevance of each temporal scale for the current emotional state, allowing the system to emphasize short-term responses during emotional transitions or long-term patterns during stable emotional states. The attention weights are computed using a multi-head attention mechanism that considers both the current feature representations and the historical context. Our proposed multi-scale temporal attention mechanism employs the following scaled dot–product attention formulation:

(1) $$\mathrm{Attention}\left(Q,K,V\right)=\mathrm{softmax}\left(\frac{QK^{T}}{\sqrt{d_{k}}}\right)V\;\;$$

where

- Q represents query vectors from current temporal features
- K: Key vectors from all temporal scales
- V: Value vectors from all temporal scales
- $d_{k}$: Dimensionality of key vectors.

The multi-scale feature fusion is computed as follows:

(2) $$F_{fused}=\sum_{s\in \{\mathrm{short},\mathrm{medium},\mathrm{long}\}}w_{s}\cdot F_{s}\;$$

- $F_{s}$: Feature representations from temporal scale s ∈ {short, medium, long}
- w_s: Learned attention weights for scale s, where Σw_s = 1
- $F_{fused}$: Final fused feature representation

Where $F_{s}$ represents features from scales∈short,medium,long and $w_{s}$ are the learned attention weights satisfying $\Sigma w_{s}$ = 1.

The adaptive fusion weights are computed using the following:

(3) $$w_{i}=\frac{\;\;\;\exp\left(g_{i}\cdot q_{i}\right)}{\sum_{j=1}^{M}\;\;\;\exp\left(g_{j}\cdot q_{j}\right)}\;$$

- M: Total number of modalities (5 in our case: EEG, PPG, GSR, Visual, and Audio)

The adaptive fusion mechanism operates through a three-stage process:

Stage 1—Quality Assessment:

(4) $$q_{i}\;=QualityAssessment\left(SNR_{i},variance_{i},missing_{r}ate_{i}\right)$$

Stage 2—Relevance Scoring:

(5) $$g_{i}=\;RelevanceNetwork\left(F_{t}emporal^{i},emotional_{c}ontext\right)$$

Stage 3—Adaptive Weight Computation:

(6) $$w_{i}=\;exp\left(g_{i}\cdot q_{i}\right)/\Sigma_{j}exp\left(g_{j}\cdot q_{j}\right)$$

where

- $q_{i}$: Quality score for modality i based on signal-to-noise ratio
- $g_{i}$: Relevance score for modality i computed through neural networks
- $w_{i}$: Final fusion weight combining both quality and relevance considerations

This three-stage process enables real-time adaptation to varying sensor conditions while maintaining emotional relevance, crucial for robust IoT deployment.

Where $g_{i}$ represents the relevance score for modality i, $q_{i}$ is the quality score, and M is the total number of modalities. This formulation enables the model to dynamically attend to the most relevant temporal features across different scales while incorporating both relevance and quality considerations in the fusion process.

Inter-modal feature fusion integrates information across different sensor modalities using a hierarchical approach. The fusion process begins with modality-specific processing that generates high-level representations for each sensor type. These representations are then combined using cross-modal attention mechanisms that learn relationships between different modalities. Cross-modal attention allows the system to focus on the most relevant modalities for the current emotional state while maintaining robustness to sensor failures or noise.

The adaptive nature of the fusion mechanism is particularly important for IoT deployment scenarios where sensor availability and quality may vary due to environmental conditions, user behavior, or device limitations. The system includes modality dropout mechanisms that can gracefully handle missing or corrupted sensor data by redistributing attention weights among available modalities. Quality assessment modules continuously monitor signal quality for each modality and adjust fusion weights accordingly.

### 3.4. EmotionTFN Training Procedure

The EmotionTFN model training follows a multi-stage optimization process designed to learn effective multimodal representations while maintaining computational efficiency for edge deployment.

The training process operates on multimodal input batches containing synchronized sensor data: EEG signals, PPG measurements, GSR readings, visual frames, and audio segments. Each modality undergoes independent preprocessing and temporal feature extraction across the three defined time scales (short-term: 0.5–2 s, medium-term: 2–10 s, and long-term: 10–60 s).

The system employs a composite loss function that balances discrete emotion classification and continuous dimensional prediction as follows:

(7) Ltotal = λ1 · Ldiscrete + λ2 · Lcontinuous + λ3 · Lregularization

where $L_{discrete}$ represents cross-entropy loss for seven-class emotion classification, $L_{continuous}$ denotes mean squared error for valence-arousal prediction, and $L_{regularization}$ includes weight decay and dropout regularization terms. The weighting parameters $\lambda_{1}=0.6,\;\lambda_{2}=0.3,\;and\;\lambda_{3}=0$ were determined through hyperparameter optimization.

Training utilizes the Adam optimizer with an initial learning rate of 0.001, employing cosine annealing with warm restarts to improve convergence stability. Batch sizes of 32 samples accommodate memory constraints while maintaining gradient stability. The adaptive fusion weights are learned jointly with the feature extractors through end-to-end backpropagation, enabling the system to automatically discover optimal modality combinations for different emotional states.

To ensure compatibility with edge deployment, training incorporates quantization-aware techniques and progressive model compression. This approach allows the model to learn robust representations that maintain accuracy even after post-training optimization for IoT devices. Early stopping based on validation performance prevents overfitting while monitoring both accuracy and computational complexity metrics.

The training process typically converges within 50–80 epochs, with model checkpoints saved based on validation performance improvements. The final trained model demonstrates robust generalization across different hardware platforms while maintaining the real-time processing capabilities essential for IoT emotion recognition applications.

### 3.5. Adaptive Fusion Mechanism

The EmotionTFN framework addresses three key technical challenges in IoT emotion recognition through integrated architectural solutions that build upon the methodologies described in the preceding sections.

The first contribution involves the integration of multi-scale temporal processing with cross-modal reliability estimation. Building upon the multi-scale temporal attention mechanism detailed in Section 3.3, this approach combines temporal feature extraction across short-term (0.5–2 s), medium-term (2–10 s), and long-term (10–60 s) windows with real-time sensor quality assessment. The system implements a feedback mechanism where sensor reliability scores influence temporal attention weight computation, while temporal context analysis informs reliability estimation accuracy. This integrated approach enables dynamic adaptation to varying sensor conditions and emotional state transitions, addressing limitations of fixed-window approaches that process temporal scales independently without quality considerations.

The second innovation, Reliability- and Latency-Aware Adaptive Sampling (RLAS), addresses the critical challenge of maintaining accuracy within strict IoT resource constraints. While existing adaptive sampling methods focus on computational load or signal quality in isolation, RLAS formulates sampling as a joint optimization problem considering latency requirements, energy consumption, and quality preservation simultaneously. This enables 98% of full-sampling accuracy with 40% energy efficiency improvements and sub-200 ms latency—a performance combination not achieved by existing methods.

The third innovation, Dynamic Multi-Modal Fusion with Quality-Adaptive Weighting (DMF-QAW), introduces automatic modality dropout compensation through correlation-based recovery mechanisms. When sensors fail, DMF-QAW leverages inter-modality relationships to redistribute attention and recover information from available sensors. This ensures graceful degradation with less than 5% accuracy loss under multiple sensor failures, compared to 10–15% degradation in existing methods.

The integration of these innovations addresses the “IoT emotion recognition trilemma”—simultaneously achieving high accuracy, real-time performance, and energy efficiency. Each component enhances the others as follows: HTA-CMRE guides RLAS decision-making, while RLAS constraints influence DMF-QAW strategies, and DMF-QAW evaluations inform HTA-CMRE mechanisms. This holistic approach demonstrates that IoT emotion recognition challenges are fundamentally interconnected and require integrated solutions rather than isolated optimizations. Algorithm 1 details the complete real-time inference procedure that implements these integrated solutions.
**Algorithm 1.** Real-time inference with adaptive sampling.Input: Sensor streams S = {sEEG, sPPG, sGSR, svisual, saudio}, trained model Output: Emotion predictions with confidence scores 1: Initialize buffers for each temporal scale 2: Initialize adaptive sampling rates rmodality for each modality 3: while system is active do 4:       current_time ← GetTimestamp() 5:       computational_load ← GetCurrentLoad() 6:       if computational_load > threshold then 7:                 sampling_rates ← AdaptiveSampling(computational_load, priority_weights) 8:       else 9:         sampling_rates ← default_rates 10:     end if 11:     12:     // Collect sensor data 13:     for each modality m ∈ {EEG, PPG, GSR, visual, audio} do 14:            if ModalityAvailable(m) then 15:                  xm ← SampleData(sm, sampling_rates[m]) 16:                  qualitym ← AssessSignalQuality(xm) 17:                  UpdateBuffer(xm, rm, current_time) 18:           end if 19:     end for 20:     21:     // Check for inference readiness 22:     if BufferSufficient() then 23:           start_time ← GetTimestamp() 24:         25:           // Multi-scale feature extraction (parallel) 26:           for each available modality m do 27:                  Fshort_m ← ExtractShortTermFeatures(bufferm, window ← 1s) 28:                  Fmedium_m ← ExtractMediumTermFeatures(bufferm, window ← 5s) 29:                  Flong_m ← ExtractLongTermFeatures(bufferm, window ← 30s) 30:         end for 31:         32:        // Adaptive fusion 33:             quality_scores ← {qualityEEG, qualityPPG, qualityGSR, qualityvisual, qualityaudio} 34:         w ← ComputeAdaptiveWeights(features, quality_scores) 35:         Ffused ← AdaptiveFusion(features, w) 36:         37:         // Emotion classification 38:         (discrete_emotions, continuous) ← Classify(Ffused, θ*) 39:         40:         // Latency check 41:         inference_time ← GetTimestamp() - start_time 42:         if inference_time > Tmax then 43:              AdjustSamplingStrategy() 44:         end if 45:         46:         // Output and logging 47:         OutputEmotionPrediction(discrete_emotions, continuous, confidence) 48:         LogPerformanceMetrics(inference_time, accuracy, energy_consumption) 49:         50:         // Update system state 51:         UpdateSensorSharedComputeLoad, sensor_status) 52:         UpdateSamplingIntervals() 53:    end if 54: end while

### 3.6. Edge Computing Optimization

Deploying complex multimodal emotion recognition systems on resource-constrained IoT devices requires optimization strategies including quantization, pruning, and adaptive sampling, that balance accuracy and computational efficiency. The proposed edge computing optimization approach includes model compression, adaptive sampling, and dynamic resource allocation techniques.

Model compression is achieved through a combination of quantization, pruning, and knowledge distillation techniques. Post-training quantization reduces the precision of model weights and activations from 32-bit floating-point to 8-bit integer representations, significantly reducing memory requirements and computational complexity. The quantization process employs calibration datasets to minimize quantization error while maintaining model accuracy.

Structured pruning removes entire channels or layers that contribute minimally to model performance while maintaining the regular structure required for efficient hardware implementation. The pruning process uses magnitude-based criteria to identify less important parameters and removes them systematically while monitoring accuracy degradation. Knowledge distillation transfers knowledge from a large teacher model to a smaller student model suitable for edge deployment. The student model is trained to mimic the output distributions of the teacher model, enabling it to achieve similar performance with significantly fewer parameters.

Adaptive sampling strategies optimize data collection based on real-time processing constraints and emotional state dynamics. The system monitors processing latency and adjusts sampling rates for different modalities to maintain real-time performance. During periods of high computational load, the system prioritizes modalities with higher discriminative power for the current emotional state. The sampling adaptation is guided by uncertainty estimates that indicate when additional data is needed for confident predictions.

### 3.7. Real-Time Emotion Classification

The final component of EmotionTFN performs real-time emotion classification using the fused multimodal features. The classification module employs a lightweight neural network architecture optimized for edge deployment while maintaining high accuracy. The network consists of fully connected layers with batch normalization and dropout for regularization.

The emotion classification scheme follows a dual approach, predicting both discrete emotion categories and continuous dimensional values.

Discrete Emotion Categories: Following Ekman’s basic emotion model, we classify emotions into the following seven categories:

1. Anger—Intense displeasure/hostility
2. Disgust—Revulsion/aversion
3. Fear—Anxiety/apprehension
4. Happiness—Joy/contentment
5. Neutral—Emotionally balanced state
6. Sadness—Sorrow/melancholy
7. Surprise—Sudden wonder/astonishment

Dimensional Model: Continuous prediction of valence (positive/negative affect, range [−1, 1]) and arousal (activation level, range [0, 1]) provides complementary emotional state representation suitable for different IoT applications. The dimensional model predicts valence (positive/negative) and arousal (high/low) values on continuous scales. This dual representation provides flexibility for different application requirements and enables more nuanced understanding of emotional states.

The classification network architecture includes three fully connected layers with dimensions 512, 256, and 128, respectively. Each layer is followed by batch normalization and dropout with a rate of 0.3 to prevent overfitting. The final layer outputs both discrete emotion probabilities through a softmax activation and continuous dimensional values through linear activations. The system provides confidence scores for each prediction, enabling downstream applications to make informed decisions about emotion-based responses.

### 3.8. Privacy and Security Considerations

The continuous collection and processing of sensitive emotional data in IoT environments raise important privacy and security concerns that must be addressed in system design. EmotionTFN incorporates several privacy-preserving mechanisms to protect user data while maintaining system functionality.

Local processing on edge devices minimizes the transmission of raw sensor data over networks, reducing exposure to potential interception or unauthorized access. When network communication is necessary, the system employs end-to-end encryption using AES-256 encryption and secure communication protocols such as TLS 1.3 to protect data in transit. Differential privacy techniques are applied to aggregated data to prevent individual identification while preserving statistical utility.

The system includes comprehensive user consent mechanisms that provide granular control over data collection and processing. Users can specify which modalities to enable, adjust privacy settings, and review data usage patterns through a transparent interface. Data retention policies ensure that sensitive information is automatically deleted after specified time periods unless explicitly retained by user request. The system maintains detailed audit logs of all data access and processing activities to ensure accountability and enable forensic analysis if necessary.

## 4. Experimental Setup

### 4.1. Datasets and Data Preparation

To evaluate the performance of EmotionTFN across diverse scenarios and modalities, comprehensive experiments were conducted on three publicly available datasets that provide multimodal emotion data suitable for IoT-based recognition systems. The selection of datasets was guided by several criteria, including availability of multiple modalities, real-world recording conditions, diverse participant populations, and compatibility with IoT deployment scenarios.

The Multimodal Emotion Lines Dataset (MELD) [21] provides conversational emotion recognition data with audio, visual, and textual modalities. The dataset contains 13,708 utterances from 1433 dialogs extracted from the television series “Friends”, with emotions labeled across seven categories: anger, disgust, fear, joy, neutral, sadness, and surprise. For the experiments, facial expression features were extracted from video frames using convolutional neural networks, and speech emotion features were extracted from audio tracks using mel-frequency cepstral coefficients and prosodic features to simulate visual and audio sensors in IoT environments.

The G-REx dataset [22] offers real-world group emotion data collected using wearable sensors during movie-watching sessions. The dataset includes electrodermal activity, photoplethysmography, and accelerometer data from 73 participants across 16 movie clips. Emotions are labeled using both discrete categories and continuous valence-arousal dimensions. This dataset is particularly relevant for IoT scenarios as it captures physiological responses in naturalistic settings with multiple participants and provides insight into group emotional dynamics.

The DEAP dataset [23] provides EEG and peripheral physiological signals recorded while participants watched music videos. The dataset includes 32-channel EEG, electromyography, galvanic skin response, respiration, plethysmography, and temperature signals from 32 participants across 40 trials. Emotions are labeled using valence, arousal, dominance, and liking dimensions on a continuous scale from 1 to 9. This dataset was used to evaluate the performance of the EEG and physiological signal processing components of EmotionTFN.

To ensure reproducible evaluation and fair comparison with existing literature, we implemented established data partitioning protocols tailored to each dataset’s characteristics. For the MELD, we used the official train/validation/test splits [24], maintaining the standardized distribution of 9989/1109/2610 utterances, respectively, to enable direct performance comparison with previous studies. The DEAP dataset evaluation employed 5-fold cross-validation with subject-independent splits, allocating 80% of participants for training and 20% for testing in each fold to ensure robust generalization across different users while preventing data leakage. Similarly, the G-REx dataset was partitioned using session-independent splits with a 60/20/20 distribution for training, validation, and testing phases, ensuring generalization across diverse emotional stimuli and participant groups. All sensor modalities were synchronized using hardware-level timestamps to maintain accurate temporal alignment, implementing a comprehensive temporal synchronization strategy that accommodates the multi-scale nature of emotional expressions across short-term (1 s), medium-term (5 s), and long-term (30 s) windows with overlapping segments.

Data preprocessing was standardized across all datasets to ensure compatibility with typical IoT sensor specifications and maintain consistency in multimodal processing. Physiological signals were resampled to common sampling rates optimized for edge deployment: EEG signals at 128 Hz, PPG signals at 64 Hz, and GSR signals at 32 Hz, with careful attention to temporal alignment throughout the resampling process. Cross-modal alignment was achieved through automatic audio-visual synchronization based on energy correlation analysis, while physiological signals were aligned using a sliding window approach to accommodate inherent latency differences between modalities. Quality control mechanisms maintained synchronization accuracy within a 25 ms tolerance threshold, with automated detection systems identifying alignment errors exceeding 50 ms for correction or exclusion from analysis. Visual data processing incorporated face alignment using 68-point facial landmark detection combined with geometric normalization to correct for rotation, scaling, and translation variations, followed by photometric normalization including histogram equalization and pixel intensity scaling to the standardized [−1, 1] range.

Audio processing employed RMS energy normalization to handle amplitude variations across different recording conditions, complemented by spectral mean and variance normalization for MFCC features to ensure consistency across diverse acoustic environments. Physiological signal normalization was tailored to each modality’s characteristics: EEG signals underwent Z-score normalization per channel with baseline correction to account for individual differences, PPG signals were processed through detrending and bandpass filtering (0.5–8 Hz) with session-wise normalization to preserve cardiovascular patterns, and GSR signals utilized min-max normalization per session to accommodate individual physiological response variations. Visual data processing leveraged the OpenFace toolkit for facial landmark extraction and expression feature computation, while audio feature extraction employed the Librosa library to capture spectral characteristics, prosodic features, and temporal dynamics essential for speech emotion recognition. These comprehensive normalization procedures ensure robust system performance across diverse IoT deployment conditions and individual user variations, providing a solid foundation for reliable emotion recognition in real-world environments.

### 4.2. Implementation Details

The EmotionTFN system was implemented using PyTorch 2.1.0 (https://pytorch.org/, accessed on 15 April 2025) with optimizations for edge deployment using TensorRT 8.6.1 (NVIDIA Corporation, Santa Clara, CA, USA) and ONNX 1.14.0 (https://onnx.ai/, accessed on 15 April 2025) frameworks. The multi-scale temporal feature extraction modules were implemented using custom CUDA 11.8 (NVIDIA Corporation, Santa Clara, CA, USA) kernels to optimize performance on GPU-enabled edge devices. Visual data processing leveraged OpenFace 2.2.0 (https://github.com/TadasBaltrusaitis/OpenFace, accessed on 15 April 2025) for facial landmark extraction and expression feature computation, while audio feature extraction employed Librosa 0.10.1 (https://librosa.org/, accessed on 15 April 2025) to capture spectral characteristics, prosodic features, and temporal dynamics essential for speech emotion recognition.

The system architecture parameters were determined through extensive hyperparameter optimization using Bayesian optimization with Gaussian process models. Key parameters include temporal window sizes with short-term windows of 1 s, medium-term windows of 5 s, and long-term windows of 30 s. The attention mechanisms employ 4 heads for intra-modal attention and 8 heads for inter-modal attention. Fusion layer dimensions are set to 256 for modality-specific processing and 512 for cross-modal fusion.

Training was performed using a distributed setup with 4 NVIDIA RTX 4060 GPUs (Nvidia, Santa Clara, CA, USA), employing mixed precision training and gradient accumulation to handle large batch sizes while maintaining numerical stability. The training process used the Adam optimizer with a learning rate of 0.001 and a weight decay of 0.0001. Learning rate scheduling was implemented using cosine annealing with warm restarts to improve convergence stability.

Edge deployment testing was conducted using NVIDIA Jetson Xavier NX (Nvidia, Santa Clara, CA, USA) and Raspberry Pi 4 devices (Raspberry, Cambridge, UK) to simulate realistic IoT hardware constraints. The Jetson Xavier NX provides 384 CUDA cores and 8 GB of memory, representing high-end IoT devices, while the Raspberry Pi 4 with 8 GB RAM represents more resource-constrained environments. Performance optimization included memory pooling, computation graph optimization, and careful scheduling of operations to minimize latency and memory usage.

### 4.3. Evaluation Metrics

The evaluation of EmotionTFN encompasses multiple dimensions relevant to IoT deployment, including recognition accuracy, computational efficiency, energy consumption, and robustness to environmental variations. Recognition accuracy was measured using standard metrics, including overall accuracy, precision, recall, and F1-score for discrete emotion classification. For dimensional emotion prediction, mean absolute error and Pearson correlation coefficients were computed to assess the agreement between predicted and ground truth values.

Computational efficiency was evaluated through comprehensive analysis of processing latency, memory usage, and throughput under different hardware configurations. Latency measurements included end-to-end processing time from sensor input to emotion prediction, with detailed breakdown analysis of preprocessing, feature extraction, fusion, and classification components. Memory usage was monitored during both training and inference phases to ensure compatibility with edge device constraints.

Energy consumption analysis was performed using specialized power measurement tools integrated with the target IoT devices. The analysis included both active processing power during emotion recognition and idle power consumption during standby periods. Adaptive sampling strategies were evaluated based on their ability to maintain recognition accuracy while reducing overall energy consumption through intelligent data collection and processing optimization.

Robustness evaluation included comprehensive stress testing under various environmental conditions, such as varying lighting conditions from 50 to 1000 lux, background noise levels from 30 to 80 decibels, and different user mobility patterns. The system’s ability to handle missing or corrupted sensor data was assessed through systematic modality dropout experiments where individual sensors were disabled or corrupted to simulate real-world failure scenarios.

### 4.4. Baseline Comparisons

To demonstrate the effectiveness of EmotionTFN, comprehensive comparisons were conducted against several state-of-the-art baseline methods representing different categories of emotion recognition systems. The baseline methods were selected to cover single-modality approaches, traditional multimodal fusion techniques, and recent deep learning-based systems.

Single-modality baselines included convolutional neural network-based facial expression recognition using ResNet-50 architecture, long short-term memory network-based speech emotion recognition with attention mechanisms, and support vector machine-based physiological signal classification using hand-crafted features. These baselines represent the performance achievable using individual sensor modalities commonly available in IoT environments.

Traditional multimodal fusion baselines included early fusion through feature concatenation, late fusion through decision-level combination using weighted voting, and intermediate fusion approaches using canonical correlation analysis. These methods provide comparison points for understanding the benefits of the proposed adaptive fusion mechanism compared to conventional fusion strategies.

Recent deep learning baselines included attention-based multimodal fusion using transformer architectures, graph neural network approaches for temporal modeling of emotional sequences, and state-of-the-art emotion recognition systems adapted for multimodal inputs. These methods represent the current state-of-the-art in multimodal emotion recognition and provide challenging comparison points for the proposed approach. Additional baselines included recent approaches such as comprehensive multimodal emotion recognition systems and extensive survey findings which provide broad coverage of deep learning-based multimodal approaches.

All baseline methods were implemented using identical preprocessing pipelines and evaluation protocols to ensure fair comparison. Hyperparameters for baseline methods were optimized using the same Bayesian optimization approach used for EmotionTFN to eliminate bias in performance comparison. The implementations were validated against published results where available to ensure correctness and reproducibility. The IEMOCAP dataset was also considered for additional validation, though the primary focus remained on the three selected datasets for consistency with IoT deployment scenarios.

## 5. Results and Analysis

### 5.1. Overall Performance Comparison

The experimental evaluation of EmotionTFN demonstrates significant improvements over existing approaches across multiple evaluation metrics and datasets. The comprehensive performance comparison reveals the effectiveness of the proposed multi-scale temporal fusion approach for real-time emotion recognition in IoT environments. Table 1. Performance comparison on multimodal emotion recognition. The comparison includes recent state-of-the-art methods: MemoCMT [25] using cross-modal transformer-based feature fusion, MIST [26] combining DeBERTa and ResNet for conversational emotion recognition, GS-MMC [27] employing graph-structured multimodal fusion, SUMMER [28] leveraging sparse mixture-of-experts with multimodal distillation, and Geetha et al. [29] providing a comprehensive deep learning framework.

Table 2 summarizes the detailed characteristics of the three datasets used in our evaluation.

Table 3 presents the hardware performance analysis across different platforms.

On the MELD, EmotionTFN achieved an overall accuracy of 94.2% for seven-class emotion classification, representing a 6.8% improvement over the best baseline method (87.4%). The performance gains were consistent across all emotion categories, with particularly notable improvements in distinguishing between subtle emotions such as neutral and sadness, where the F1-score improved from 0.78 to 0.91, and between fear and surprise, where the F1-score improved from 0.72 to 0.88.

The G-REx dataset evaluation focused on physiological signal-based emotion recognition in naturalistic settings. EmotionTFN achieved 89.7% accuracy for valence classification and 91.3% accuracy for arousal classification, outperforming the best baseline by 7.2% and 5.9%, respectively. The correlation coefficients for continuous emotion prediction were 0.847 for valence and 0.863 for arousal, demonstrating strong agreement with human annotations and indicating the system’s ability to capture subtle emotional variations.

DEAP dataset results demonstrated the effectiveness of the multi-scale temporal modeling approach for EEG-based emotion recognition. The system achieved mean absolute errors of 0.087 for valence and 0.094 for arousal prediction, representing 23% and 19% improvements over the best baseline methods. The temporal analysis revealed that the multi-scale approach was particularly effective for capturing both rapid emotional responses occurring within seconds and longer-term mood patterns that persist over minutes.

### 5.2. Ablation Study

To understand the contribution of different components in EmotionTFN, comprehensive ablation studies were conducted that systematically removed or modified key architectural elements. The ablation study provides insights into the importance of each component and validates the design decisions made in the proposed architecture. Table 4 shows the results of our comprehensive ablation study.

The multi-scale temporal modeling component showed the largest individual contribution to system performance. Removing this component and using fixed-window processing resulted in accuracy drops of 8.3%, 6.7%, and 5.7% on MELD, G-REx valence, and G-REx arousal, respectively. For the DEAP dataset, the mean absolute error increased significantly from 0.087 to 0.121 for valence and from 0.094 to 0.127 for arousal. This confirms the critical importance of capturing emotional dynamics across multiple temporal scales for effective emotion recognition.

The adaptive fusion mechanism contributed 4.4% to 4.2% accuracy improvements across datasets compared to configurations without adaptive fusion. Replacing adaptive fusion with simple concatenation or fixed-weight combination significantly degraded performance, particularly for scenarios with varying signal quality or missing modalities. The cross-modal attention component within the fusion mechanism was responsible for approximately 60% of the fusion-related performance gains, highlighting the importance of learning relationships between different sensor modalities.

Edge computing optimizations, while primarily designed for computational efficiency, also contributed to accurate improvements through regularization effects. The optimized configuration achieved only 0.4% accuracy loss compared to the full model while reducing processing latency by 40%. Quantization and pruning techniques resulted in minimal accuracy degradation while achieving significant computational savings. The adaptive sampling component maintained 98% of full-sampling accuracy while reducing computational load by 35%.

### 5.3. Computational Efficiency Analysis

The computational efficiency evaluation demonstrates EmotionTFN’s suitability for real-time IoT deployment across different hardware configurations. The analysis reveals the system’s ability to maintain high performance while operating within the constraints of resource-limited edge devices. The computational efficiency analysis is detailed in Table 5.

On NVIDIA Jetson Xavier NX, representing high-end IoT devices, the end-to-end processing latency averaged 187 ms, well below the 200 ms target for real-time applications. The latency breakdown analysis revealed that feature extraction consumed 45% of processing time, fusion operations required 30%, and classification utilized 25% of the total processing time. Memory usage peaked at 2.1 GB during inference, well within the 8 GB capacity of the target device.

Raspberry Pi 4 evaluation, representing resource-constrained IoT devices, achieved 298 ms average latency with the optimized model configuration. While exceeding the strict real-time threshold, this performance is acceptable for many IoT applications that do not require immediate response, such as mood monitoring or long-term emotional trend analysis. Memory usage was optimized to 1.8 GB through aggressive model compression and memory pooling techniques.

The adaptive sampling mechanism demonstrated significant efficiency improvements under varying computational loads. During high-load periods, the system automatically reduced sampling rates for less critical modalities while maintaining 92% of full-performance accuracy. Energy consumption analysis showed a 40% reduction in power usage during adaptive sampling periods, extending battery life in portable IoT devices.

### 5.4. Multi-Scale Temporal Analysis

The multi-scale temporal modeling approach represents a key innovation of EmotionTFN, enabling the system to capture emotional dynamics across different time scales simultaneously. Detailed analysis of the temporal attention patterns reveals how the system adapts to different emotional expression characteristics. Table 6 shows the temporal attention weight patterns for different emotions.

The temporal attention analysis reveals distinct patterns for different emotional states. Surprise shows the highest attention weight for short-term features (0.51), reflecting the sudden onset characteristic of surprise reactions. Conversely, neutral and sad states show higher attention weights for long-term features (0.37), indicating that these states are better characterized by sustained patterns rather than immediate responses.

The system’s ability to adapt temporal attention based on emotional context is demonstrated through dynamic attention weight changes during emotional transitions. During periods of emotional stability, long-term attention weights increase to capture sustained mood patterns. During emotional transitions, short-term attention weights increase to capture rapid changes in physiological and behavioral indicators.

Robustness evaluation assessed EmotionTFN’s performance under realistic IoT deployment conditions including environmental variations, sensor failures, and cross-user generalization. The system maintains > 85% accuracy under sensor failures, confirming its suitability for real-world deployment. The robustness evaluation results are summarized in Table 7.

Environmental stress testing included variations in lighting conditions from 50 to 1000 lux, background noise levels from 30 to 80 decibels, and different user mobility patterns. Performance degradation was minimal across all tested conditions, with accuracy drops of less than 3% in most scenarios. The system’s robustness to environmental variations is attributed to the multimodal fusion approach and adaptive quality assessment mechanisms.

Sensor failure simulation evaluated the system’s ability to handle missing or corrupted modalities through systematic dropout experiments. With single modality failures, accuracy degradation ranged from 2.1% for audio sensor failure to 4.7% for EEG sensor failure. The differential impact reflects the varying importance of different modalities for emotion recognition, with EEG providing unique neural activity information that is difficult to compensate for from other sensors.

Even with two simultaneous modality failures, the system maintained over 85% of baseline performance through the adaptive fusion mechanism. The system’s graceful degradation capability is crucial for IoT deployment scenarios where sensor reliability may be compromised due to environmental conditions, battery depletion, or hardware failures.

### 5.5. Real-World Deployment Case Study

To validate EmotionTFN’s practical applicability, a comprehensive real-world deployment study was conducted in a smart home environment with 12 participants over a 4-week period. The deployment included wearable sensors integrated into smartwatches for PPG and GSR monitoring, ambient cameras for facial expression analysis, and smart speakers for audio emotion recognition. Table 8 presents the real-world deployment results over the four-week period.

The system successfully operated continuously with 97.2% average uptime over the four-week period. System failures were primarily attributed to network connectivity issues and occasional sensor calibration drift rather than fundamental system malfunctions. The deployment demonstrated the system’s reliability for long-term continuous operation in real-world conditions.

Average processing latency in the real deployment was 206 ms, slightly higher than laboratory conditions due to network communication overhead and environmental interference. However, the latency remained within acceptable bounds for most emotion-aware applications. User acceptance evaluation showed progressive improvement in satisfaction scores from 3.8 to 4.2 over the deployment period, indicating successful user adaptation and system refinement.

Privacy concerns were minimal and decreased over time, with average scores of 1.8 on a scale where 1 indicates no concerns and 5 indicates major concerns. The low privacy concern ratings are attributed to the system’s local processing capabilities, transparent data handling policies, and comprehensive user control mechanisms.

The deployment study revealed several practical considerations for IoT emotion recognition systems. Sensor placement and calibration significantly impact performance, requiring careful attention during installation and periodic recalibration. Environmental factors such as lighting changes and background noise require adaptive algorithms to maintain consistent performance. User behavior patterns vary significantly across individuals, highlighting the importance of personalization and adaptation mechanisms.

## 6. Discussion

### 6.1. Technical Contributions and Significance

The Multi-Scale Temporal Fusion Network represents a significant advancement in the field of IoT-based emotion recognition through several key technical contributions. The most important innovation is the multi-scale temporal modeling approach that simultaneously captures emotional dynamics across different time scales. This capability addresses a fundamental limitation of existing emotion recognition systems that typically operate on fixed time windows and fail to capture the complex temporal nature of human emotions.

The hierarchical temporal attention mechanism enables the system to adaptively focus on different time scales based on the current emotional context. This adaptation is crucial for real-world applications where emotional expressions vary significantly in their temporal characteristics. Rapid emotional responses such as surprise or fear require immediate detection through short-term features, while sustained emotional states such as sadness or contentment are better characterized through long-term patterns.

The adaptive fusion mechanism represents another significant contribution, addressing the challenge of integrating multimodal sensor data in dynamic IoT environments. Unlike traditional fusion approaches that use fixed combination strategies, the proposed adaptive fusion learns to weigh different modalities based on their current reliability and relevance to the emotional state. This capability is essential for robust operation in IoT environments where sensor availability and quality may vary due to environmental conditions or hardware limitations.

The comprehensive edge computing optimization approach demonstrates that complex multimodal emotion recognition can be successfully deployed on resource-constrained IoT devices without significant accuracy degradation. The combination of model compression, adaptive sampling, and dynamic resource allocation provides a template for deploying other computationally intensive artificial intelligence applications in IoT environments.

### 6.2. Implications for IoT Emotion Recognition

The results of this study have far-reaching implications for the development and deployment of emotion recognition systems in IoT environments. The demonstrated ability to achieve high accuracy while maintaining real-time performance on resource-constrained devices opens new possibilities for emotion-aware IoT applications across multiple domains.

In healthcare applications, continuous emotion monitoring can provide valuable insights into patient mental health and treatment effectiveness. The non-intrusive nature of the proposed approach, combined with privacy-preserving processing, addresses key concerns about patient acceptance and data protection. The system’s robustness to environmental variations makes it suitable for home-based monitoring scenarios where controlled laboratory conditions are not feasible.

Smart home applications can benefit from emotion-aware automation that adapts to residents’ emotional states and preferences. The multi-scale temporal modeling capability enables the system to distinguish between momentary emotional responses and longer-term mood patterns, allowing for appropriate automation responses. Brief frustration might trigger immediate assistance, such as adjusting lighting or playing calming music, while persistent sadness could prompt longer-term environmental adjustments or suggestions for social interaction.

Human–computer interaction applications can leverage real-time emotion recognition to create more natural and responsive interfaces. The low latency and high accuracy of EmotionTFN enable immediate adaptation of interface behavior based on user emotional state, improving user experience and task performance. Educational applications can adapt content difficulty and presentation style based on student emotional engagement, while entertainment systems can adjust content recommendations based on mood preferences.

### 6.3. Why EmotionTFN Represents a Fundamental Advancement

The technical contributions of EmotionTFN extend beyond incremental improvements to represent an advancement in IoT-based emotion recognition. While existing systems typically address individual challenges (temporal modeling, sensor fusion, or edge optimization) in isolation, our integrated approach demonstrates that these challenges are fundamentally interconnected and require joint solutions.

The novelty lies not only in the individual technical components but in their synergistic integration: HTA-CMRE’s reliability assessment informs RLAS decisions, which in turn affects DMF-QAW fusion strategies, all optimized within the edge computing framework. This holistic approach addresses the “IoT emotion recognition trilemma”—the need to simultaneously achieve high accuracy, real-time performance, and energy efficiency under dynamic conditions.

Our comprehensive validation demonstrates that this integrated approach achieves performance levels not attainable through combining existing methods, confirming the fundamental nature of our technical contributions to the field of affective computing in IoT environments.

### 6.4. Limitations and Future Directions

While EmotionTFN demonstrates significant improvements over existing approaches, several limitations remain that present opportunities for future research. The current system requires initial calibration and training data for optimal performance, which may limit its applicability in scenarios where such data is not readily available. Future work could explore few-shot learning and meta-learning approaches to reduce data requirements for new deployments and enable rapid adaptation to new users or environments.

The evaluation was conducted primarily on established datasets and controlled deployment scenarios. More extensive real-world validation across diverse populations, cultures, and environmental conditions would strengthen the evidence for practical applicability. Long-term longitudinal studies could provide insights into system performance over extended deployment periods and user adaptation patterns.

Cultural variations in emotional expression represent an important consideration for global deployment of emotion recognition systems. The current evaluation included participants from multiple demographic groups, but more extensive validation is needed to ensure fair and accurate performance across different cultural backgrounds and expression patterns. Future research should investigate cultural adaptation mechanisms and develop culturally aware emotion recognition models.

The current emotion model focuses on basic emotions and dimensional representations commonly used in affective computing research. Future extensions could incorporate more sophisticated emotion models that capture complex emotional states such as mixed emotions, emotional transitions, and culturally specific emotional concepts. Integration with cognitive and social psychology theories could provide deeper insights into emotional processes and improve recognition accuracy.

Privacy and security considerations, while addressed in the current design, require ongoing attention as new threats and regulations emerge. Future work could explore advanced privacy-preserving techniques such as federated learning, homomorphic encryption, and differential privacy to further enhance data protection while maintaining system functionality. The development of standardized privacy frameworks for emotion recognition systems would benefit the entire field.

### 6.5. Broader Impact and Ethical Considerations

The deployment of emotion recognition systems in IoT environments raises important ethical considerations that must be carefully addressed to ensure responsible development and deployment. Continuous monitoring of emotional states has the potential for both beneficial applications and harmful misuse, requiring clear guidelines and regulations to protect user interests.

Important considerations for future work include addressing potential bias across diverse populations and understanding the psychological impact of continuous emotion monitoring on users. Ethical guidelines and user-centered design principles are essential for responsible deployment.

Data ownership and control represent critical issues for IoT emotion recognition systems. Users must have a clear understanding and control over how their emotional data is collected, processed, stored, and used. The development of standardized privacy frameworks and user interfaces for emotional data management is essential for widespread adoption of these technologies.

The potential for misuse of emotion recognition technology by malicious actors or authoritarian governments raises serious concerns about surveillance and social control. Technical safeguards, legal protections, and international cooperation are needed to prevent abuse while enabling beneficial applications of the technology.

## 7. Conclusions

This paper presents the Emotion-aware Multi-Scale Temporal Fusion Network (EmotionTFN), a novel architecture for real-time multimodal emotion recognition specifically designed for IoT environments. The proposed system addresses key challenges in IoT-based emotion recognition through innovative multi-scale temporal modeling, adaptive fusion mechanisms, and comprehensive edge computing optimizations.

The experimental evaluation demonstrates significant improvements over existing approaches, with EmotionTFN achieving 94.2% accuracy on emotion classification while maintaining processing latency below 200 ms on typical IoT hardware. The system shows robust performance across diverse environmental conditions and demonstrates practical applicability through real-world deployment validation. The comprehensive ablation study confirms the importance of each system component and validates the design decisions made in the proposed architecture.

The key contributions of this work include the development of a multi-scale temporal fusion architecture that captures emotional dynamics across different time scales, the implementation of adaptive fusion mechanisms that maintain performance under varying sensor conditions, the design of comprehensive edge computing optimizations that enable real-time processing on resource-constrained devices, and the provision of extensive evaluation demonstrating practical applicability for IoT deployment.

The implications of this research extend beyond technical contributions to broader considerations of ethical deployment, user privacy, and social impact. The demonstrated feasibility of accurate, real-time emotion recognition in IoT environments opens new possibilities for emotion-aware applications while raising important questions about responsible development and deployment practices.

Future research directions include extending the emotion model to capture more complex emotional states, developing privacy-preserving techniques for distributed emotion recognition, investigating cultural adaptation mechanisms for global deployment, and conducting long-term studies to understand the impact of continuous emotion monitoring on user behavior and well-being.

The work presented in this paper represents a significant step toward practical, accurate, and privacy-preserving emotion recognition in IoT environments. The comprehensive evaluation and real-world deployment validation provide evidence for the system’s readiness for practical applications, while the identification of limitations and future research directions provides a roadmap for continued advancement in this important field.

The open-source implementation of EmotionTFN will be made available to facilitate reproducible research and accelerate the development of emotion-aware IoT applications. The research community is encouraged to build upon this work and explore new applications and improvements to advance the field of affective computing in IoT environments.

## Figures and Tables

**Table 1:** Performance comparison on multimodal emotion recognition (updated).

Method	MELD Acc. (%)	DEAP MAE (V/A)	G-REx Acc. (V/A %)	Avg. Acc. (%)	Latency (ms)
MemoCMT (Cross-modal Transformer)	91.3	–	–	91.3	250
MIST (DeBERTa+ResNet Fusion)	88.7	–	–	88.7	240
GS-MMC (Graph-Structured Fusion)	85.4	–	–	85.4	220
SUMMER (Sparse MoE Distillation)	83.0	–	–	83.0	230
EmotionGPT (LLM-based)	78.2	0.115/0.121	78.9/81.2	79.4	285
Geetha et al. (Deep Learning)	75.2	0.125/0.135	76.8/78.9	76.9	160
Bang et al. (Hybrid Framework)	73.1	0.138/0.145	74.2/76.5	74.6	145
Shi et al. (Music-inspired)	71.8	0.142/0.152	72.5/75.1	73.1	135
Yi et al. (Hypergraph)	70.3	0.156/0.164	71.2/73.8	71.8	165
EmotionTFN (Proposed)	94.2	0.087/0.094	89.7/91.3	91.8	187

**Table 2:** Detailed dataset characteristics.

Dataset	Modalities	Participants	Samples	Duration	Emotion Labels	IoT Relevance
MELD	Audio, Video, Text	1433	13,708	~24 h	7 discrete	Conversational
DEAP	EEG(32ch), PPG, GSR	32	1280	63 min	4 dimensional	Physiological
G-REx	EDA, PPG, ACC	73	1168	32 min	2 dimensional	Wearable sensors

**Table 3:** Hardware performance analysis.

Platform	CPU	Memory (GB)	GPU	Latency (ms)	Throughput (FPS)	Power (W)	Energy (J/inf)
RTX 4060	-	4.2	10,496 CUDA	23	43.5	320	7.36
Jetson Xavier NX	ARM64	2.1	384 CUDA	187	5.3	15	2.81
Raspberry Pi 4	ARM64	1.8	-	298	3.4	8	2.68
Jetson Nano	ARM64	1.2	128 CUDA	445	2.2	5	2.23

**Table 4:** Ablation study results.

Configuration	MELD Acc (%)	G-REx Val (%)	G-REx Aro (%)	DEAP Val (MAE)	DEAP Aro (MAE)	Latency (ms)
Full EmotionTFN	94.2	89.7	91.3	0.087	0.094	187
w/o Multi-scale	85.9	83.0	85.6	0.121	0.127	145
w/o Adaptive Fusion	89.8	85.5	87.1	0.098	0.104	156
w/o Cross-modal Att	91.4	87.2	89.5	0.092	0.098	168
w/o Edge Optimization	93.8	89.1	90.7	0.089	0.095	312
Fixed Windows	88.1	84.3	86.8	0.105	0.112	163
Single Modality Best	76.5	75.2	78.1	0.135	0.142	89

**Table 5:** Computational efficiency analysis.

Hardware Platform	Latency (ms)	Memory (GB)	Power (W)	Throughput (FPS)	Energy per Inference (J)
NVIDIA RTX 3090	23	4.2	320	43.5	7.36
Jetson Xavier NX	187	2.1	15	5.3	2.81
Raspberry Pi 4	298	1.8	8	3.4	2.68
Jetson Nano	445	1.2	5	2.2	2.23

**Table 6:** Temporal scale attention weights by emotion.

Emotion	Short-Term (0.5–2 s)	Medium-Term (2–10 s)	Long-Term (10–60 s)
Anger	0.42	0.38	0.20
Disgust	0.38	0.35	0.27
Fear	0.45	0.33	0.22
Happiness	0.32	0.41	0.27
Neutral	0.28	0.35	0.37
Sadness	0.25	0.38	0.37
Surprise	0.51	0.31	0.18

**Table 7:** Robustness analysis results.

Condition	Accuracy Drop (%)	Latency Impact (ms)	Recovery Time (s)
Low Light (50 lux)	2.1	+12	0.8
High Noise (80 dB)	2.8	+8	1.2
User Movement	1.9	+15	0.5
Single Sensor Failure	2.1–4.7	−23	0.3
Two Sensor Failures	6.2–9.1	−45	0.7
Network Latency	0.3	+34	2.1

**Table 8:** Real-world deployment results.

Metric	Week 1	Week 2	Week 3	Week 4	Average
System Uptime (%)	95.2	97.8	98.1	97.6	97.2
Accuracy (%)	91.3	92.1	92.8	92.4	92.2
Avg. Latency (ms)	215	208	203	198	206
User Satisfaction (1–5)	3.8	4.1	4.3	4.2	4.1
Privacy Concerns (1–5)	2.1	1.8	1.6	1.7	1.8

## Data Availability

The public datasets used in this study are available as follows: MELD is available at https://affective-meld.github.io/ (accessed on 15 April 2025) under academic license; DEAP dataset is available at https://www.eecs.qmul.ac.uk/mmv/datasets/deap/ (accessed on 30 April 2025) upon request to the original authors; G-REx dataset is available through the original publication repositories. The real-world deployment data collected during the case study cannot be shared publicly due to privacy regulations and participant consent restrictions. The EmotionTFN implementation code and preprocessing scripts are available at https://github.com/SungwookYoon/1mstfn (accessed on 30 April 2025) under MIT license. An audio overview is available at https://notebooklm.google.com/notebook/5971f1fb-d25e-4dc9-8f35-b1182869b4be/audio (accessed on 30 April 2025). Researchers interested in accessing specific experimental configurations or additional analysis code may contact the corresponding author with reasonable requests.
